# Socio-economic drivers of irrigated paddy land abandonment and agro-ecosystem degradation: Evidence from Japanese agricultural census data

**DOI:** 10.1371/journal.pone.0266997

**Published:** 2022-04-14

**Authors:** Kota Mameno, Takahiro Kubo

**Affiliations:** 1 Department of Resource and Environmental Economics, Graduate School of Agricultural Science, Tohoku University, Sendai, Miyagi, Japan; 2 Biodiversity Division, National Institute for Environmental Studies, Tsukuba, Ibaraki, Japan; 3 School of Anthropology and Conservation, University of Kent, Canterbury, United Kingdom; 4 Department of Zoology, University of Oxford, Oxford, United Kingdom; Institute for Advanced Sustainability Studies, GERMANY

## Abstract

The abandonment of irrigated paddy land has increased in Japan, which can cause a decline in food security and biodiversity. Despite the importance of individual decisions, most studies have only examined regional or community-based determinants of paddy land abandonment. This study aimed to uncover the socio-economic determinants affecting individual landowners’ decisions to abandon paddy land, using Japanese agricultural census data (2005, 2010, and 2015) composed of over one million unique paddy landowners. Results showed that low agricultural benefits are a key driver of abandonment, similar to European countries. Conversely, there is a positive correlation between the population of full-time cultivators in a household and paddy land abandonment, which contradicts previous evidence. Although some mosaics of socio-ecological landscapes with high biodiversity formed through long-term human influence (i.e., the Satoyama landscapes) are less-favored agricultural areas, the paddy land in some of these landscapes tends not to be abandoned. These findings support effective policymaking that balances biodiversity conservation and the provision of agroecosystem services in semi-natural landscapes.

## Introduction

Irrigated paddy land is a key component of private land in the Asian monsoon climate region, accounting for over 60% of the world’s irrigated farmland [1,2]; sustainable paddy land cultivation plays an essential role in both food security and biodiversity conservation [3]. This is because paddy land provides refuge for species dependent on aquatic and wetland ecosystems as a substitute for natural aquatics and wetlands [4,5]. Furthermore, sustainable paddy land cultivation also provides some benefits to human society, including beautiful landscapes (e.g., [6,7]), natural hazard prevention (e.g., [8,9]), water purification (e.g., [10]), prevention of flood and soil erosion (e.g., [10,11]), and mitigation of climate change (e.g., [12]).

In contrast, paddy land has been abandoned as well as other types of farmlands around the world, especially in developed countries [13]. This trend can continue in developing countries that are undergoing rapid socio-economic changes [14]. This is a significant challenge in terms of both biodiversity conservation and food security [13] since irrigated paddy land abandonment significantly damages amphibians, fish [15], birds [16], grassland plant species [17], and herbivores such as insects [18]. Rewilding and passive landscape restoration could decrease biodiversity rather than increase it in an Asian monsoon climate region [19], whereas recent farmland abandonment is recognized as an opportunity to restore ecosystems and biodiversity through the practice of rewilding in European and Oceanian countries [20,21]. Therefore, conservation strategies for paddy lands should be developed considering regional differences.

However, there is limited knowledge regarding the landowners’ determinants of irrigated paddy land abandonment in the Asian monsoon climate region. First, most studies were conducted in China, which mainly cultivated corn and wheat in drylands and grasslands [22–26]. Second, most studies used community-based agricultural data instead of individual landowner behavior [27] although individual decision-making is a key component of private land use. Considering that this region’s unique cultivation system provides wetland habitats and accounts for 75% of the global rice production [16,28], it is essential to understand the individual determinants of irrigated paddy land abandonment to balance biodiversity conservation and food security.

**Fig 1:**
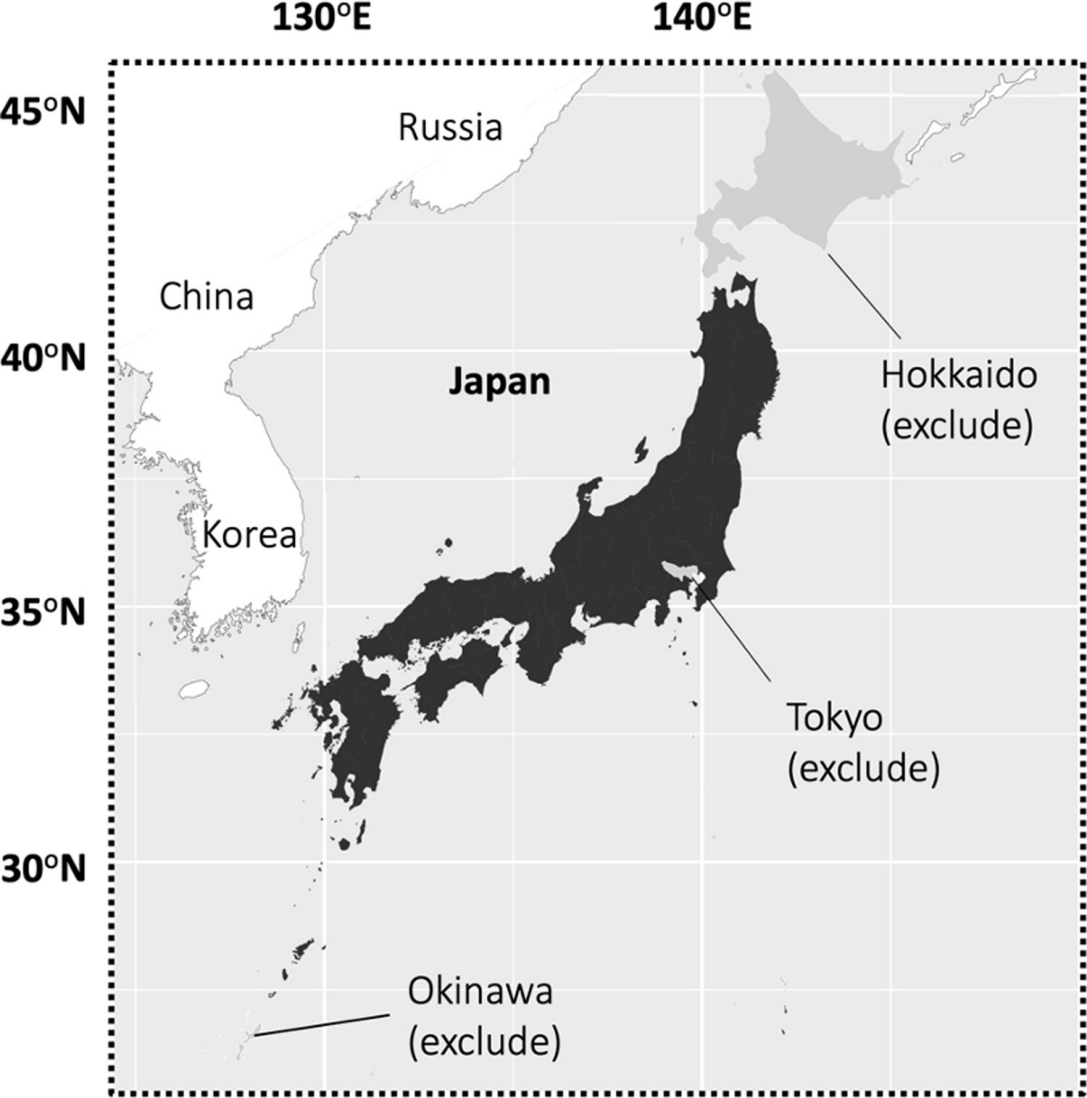
Map of Japan indicating study site regions. Japan is indicated in black and gray: Black shows where the data for the analysis were gathered; the gray areas show where the data were excluded, that is, Hokkaido, Tokyo, and Okinawa. This map was created based on Natural Earth data (http://www.naturalearthdata.com/).

The purpose of this study is to describe the individual socio-economic determinants affecting irrigated paddy land abandonment in the Asian monsoon region using the Japanese irrigated paddy land abandonment case. Japan is located in eastern Asia (see Fig 1) and is a major rice-producing country. Over half of the Japanese farmland is covered with irrigated paddy fields, and the paddy land area is approximately 30 times larger than the natural wetland area (Geospatial Information Authority of Japan: https://www.gsi.go.jp). Irrigated paddy land provides habitat for more than five thousand species; however, many species using paddy lands have been threatened [10,29]. A total of 32 species of 135 avian species, for example, have been reported as endangered species [29]. Even worse is the fact that the recent changes in socio-economic structure (e.g., urbanization and development of industries apart from agriculture) have caused paddy land abandonment in Japan, similar to the abandonment of dry farmland in Europe and the United States [30,31].

Approximately 40% of Japanese farmland, including irrigated paddy land, is located in less-favored areas, which are defined as areas with natural handicaps and/or mountainous areas [31]. Less-favored areas contribute to mosaic socio-ecological landscapes (i.e., Satoyama landscapes), and thus biodiversity conservation, although they tend to provide lower yields in paddy land compared to plain lands. Therefore, the Japanese government introduced some unique subsidies for local communities to maintain paddy land in less-favored areas [32]. The direct payment scheme for less-favored areas is an example that was introduced in 2000 to compensate for the gap in production costs between less-favored and flat areas. The scheme is based on Japanese laws concerning less-favored areas (e.g., the mountainous area development law), and rural communities with sloped paddy lands located in target regions can receive subsidies. The target areas are composed of some regions, including forestry regions with insufficient public service provision (hereinafter called Forestry Areas), rural regions in prominent depopulated areas (hereinafter called Depopulated Areas), and rural regions in mountainous and/or hilly areas (hereinafter called Mountain Areas). In other words, farmers can only receive subsidies if both communities are located in the target region and the paddy land is located in steep areas. These subsidy policies have been discussed in agricultural economics (see Literature Review for details), and few studies have focused on mechanisms to maintain paddy land in less-favored areas in the context of conservation. In light of the above research gap, the present study contributes to both biodiversity conservation and sustainable paddy land cultivation through Japanese census data analysis.

### Literature review

Farmland abandonment, including paddy land abandonment, has both positive and negative effects on biodiversity richness. A review of the literature shows that the impacts of farmland abandonment on biodiversity have notable regional differences as well as differences due to the target species [13]. Many studies have reported positive impacts of abandonment on biodiversity in Central and South America; however, in Asia, including Asian monsoon regions, negative impacts on biodiversity have been reported more than positive impacts [13]. A recent review revealed that farmland abandonment caused a 50–70% decrease in biodiversity richness in the Asian monsoon regions. In particular, given that mosaic landscapes, including irrigated paddy land, provide high biodiversity (i.e., Satoyama landscapes), paddy land abandonment significantly threatens biodiversity, such as wetland specialist species [15]. In addition to biodiversity, farmland abandonment can affect agroecosystem services, although review studies imply that few valuation studies address the impacts of farmland abandonment [33]. Given the multiple agroecosystem services in the Satoyama landscape, paddy land abandonment located in less-favored areas can cause the loss of human welfare through the degradation of beautiful landscapes, water quality, and the increase in natural hazards, floods, and soil erosion [10].

To prevent farmland abandonment, drivers have been identified to affect changes in farmland use, such as abandonment in Europe, North and South America, and Oceania, especially in developed countries [9,13,14,33–35]. For example, a literature review uncovered three main drivers, and socio-economic aspects are one of the key drivers affecting farmland abandonment [35]; the socio-economic aspects, for example, include market incentives and characteristics of farmers, including age and education level [35]. Moreover, there is literature which indicates the importance role of women in sustainable cultivation [36–38]. Evidence suggests that farmland’s location is an important factor affecting land abandonment. For example, farmland located in marginal areas with relatively poor biophysical and topographical conditions for agriculture, such as climate and slope, tends to be abandoned [33,35,39]. In these areas, monocultural cropping is adopted because farmers choose to plant only high productivity crops so they can benefit and generate good earnings [9]. In light of this evidence, some governments have introduced measures such as EU direct payment schemes to prevent farmland abandonment [14]. Furthermore, it has attracted attention to a market-based approach that incentivizes sustainable farmland cultivation [40,41]. The market-based approach includes additional payments from consumers to maintain non-material benefits from farmland when they buy an agricultural product.

There are only a few studies that address the socio-economic drivers affecting changes in Japanese paddy land use, although Japan, much like Europe and the United States, has also experienced extensive paddy land abandonment [34]. In the limited studies, there are two primary streams of research on farmland abandonment: understanding the mechanisms or drivers (e.g., [27,42]) and evaluating the impact of measures that prevent farmland abandonment (e.g., [30,43]). Interestingly, regarding the former, recent literature has found that irrigated paddy land is abandoned more than any other type of farmland in Japan [42]. Moreover, this study uncovered that a shortage of laborers and successors is related to the abandonment of farmland, including irrigated paddy land [42]. Another study revealed that the higher the ratio of elderly people in the region, the more abandoned farmland [27]. Furthermore, Kamada and Nakagoshi showed that mountainous farmland was abandoned and transformed into forest plantations by using two rural cases [44], and other previous studies also revealed similar evidence that paddy lands located in less-favored areas tended to be abandoned (in a Japanese journal) [45,46]. This trend is similar to other developed East Asian countries such as China and Korea [34]. Yoshida et al. revealed that diverse regional drivers affect farmland abandonment [46]. However, these studies analyzed community-based agricultural data, but only limited studies addressed the drivers affecting land abandonment using individual data: Senda [47], Hattori and Yamaji [48], and Kurihara et al. [49]. For example, Senda [47] and Kurihara et al. [49] explored the drivers of farmland abandonment by using the data from the Japanese Census of Agriculture and Forestry as well as the present study; on the other hand, Hattori and Yamaji employed a questionnaire survey to identify the main preventions to sustainable cultivation [48]. Although the studies provided insightful knowledge, they analyzed data from limited regions and single-year data rather than panel data.

There are some studies and reports concerning Japanese direct payment schemes for farmland abandonment, although the research topic has low relevance to the main subject of this study. In Japan, the direct payment scheme for less-favored areas was introduced to conserve multiple agroecosystem services through sustainable paddy land cultivation. Of the rural communities that can receive subsidies, 72% of the rural communities received them [50]. The direct payment scheme for less-favored areas requires the cooperation of community members since 2011 because of the importance of common-pool resources, such as irrigation and farm roads for paddy cultivation [51]. Common-pool resources have long been collectively managed by farmers in rural communities [52]. As a result of the direct payment scheme for less-favored areas, recent studies have uncovered a decrease in farmland abandonment, including paddy land, by using causal impact evaluation methods [30,43,53]. For example, Takayama and his colleagues showed a 5–8% decrease in farmland abandonment due to the direct payment scheme for less-favored areas by using individual agricultural census data [53].

## Materials and methods

### Dataset and data description

This study analyzed balanced panel data of individual irrigated paddy landowners and their households taken from the Japanese Census of Agriculture and Forestry, which is a questionnaire survey of all households owning farmland and forestland. This census has been conducted every five years by the Ministry of Agriculture, Forestry, and Fisheries (MAFF) since 1950. Our data includes three periods: 2005, 2010, and 2015. In our study, the data of paddy landowners living in Hokkaido Prefecture, Tokyo Prefecture, and Okinawa Prefecture were not included (see Fig 1) because they had different types of general agriculture systems [27]. We also excluded incomplete panel data (i.e., unconnected data from 2005 to 2010 and 2015). Finally, the completed panel data of 1,590,036 individual paddy landowners (i.e., 1,590,036×3 = 4,770,108 samples) were analyzed in our study. Our dataset includes non-farmer landowners; therefore, it can be different from the statistical data presented by the government. Previous studies have shown the importance of paddy land location as a determinant of farmland abandonment [45,46]. Therefore, the characteristics of the regions where paddy land was located were included in Model 2. These data were obtained from the Rural Community Survey of the Census of Agriculture and Forestry in Japan. The survey included whether paddy land is located in the following three regions: Forestry Areas, Depopulated Areas, and Mountain Areas. These regions are recognized as some of the less-favored areas in Japan and are target regions of the direct payment scheme despite the inclusion of uncovered paddy lands by the scheme [31]. We also included the variable *Agri-Prom*, which refers to whether the government promoted agriculture in the region. The number of samples used in Model 2 was lower than that of samples in Model 1, and there were 2,532,247 samples in total because some individual samples were not connected with community-level data.

Referring to previous studies [34,35], eighteen explanatory variables were selected. Definitions of the key variables are listed in Table 1. The dependent variable was the amount of irrigated paddy land abandoned; Table 1 presents the descriptive statistics of the variables. The findings show that the mean of the abandoned paddy land area has increased in the past decade and that 927 ㎡ of paddy lands have been abandoned per farm household in Japan from 2010 to 2015. Although the mean landowner age has decreased, the average age of household members has increased. Table 1 also shows a decrease in the number of household members under 14 years of age and the number of farmers who mainly produce rice. Moreover, although we have excluded from the analysis to avoid multicollinearity in this study, we note that about 6048 and 5155 ㎡ are the mean areas of potentially cultivable paddy land per farm household in 2010 and 2015, respectively; thus, it also decreased the mean areas of potentially cultivable paddy land from 2010 to 2015.

**Table 1:** The descriptions of the variables used in Tobit models.

Variables	Definition	2005	2010	2015
		Mean (StDev)		
*Aband-Pad*	The area of paddy land abandonment (a)	3.614 (15.02)	3.165 (12.62)	4.092 (16.80)
*Own-Age*	The age of the landowner	63.80 (12.02)	54.44 (26.71)	54.95 (27.96)
*Own-Day*	The days that the landowner cultivates	4.285 (2.307)	3.215 (2.930)	2.567 (2.927)
*Workers*	The population of cultivators in a household	3.593 (1.678)	2.371 (2.098)	1.766 (1.991)
*Pro-Fam*	The population of full-time cultivators in a household	0.2747 (0.5831)	0.9980 (1.107)	0.7828 (1.042)
*Ave-Age*	The average age of each household	56.38 (11.01)	58.60 (11.19)	60.67 (11.23)
*Woman*	The rate of women in the household	0.5029 (0.1694)	0.4974 (0.1749)	0.4876 (0.1845)
*Yang*	The population of people who are under 14 years old in a household	0.4266 (0.8576)	0.2455 (0.6822)	0.1510 (0.5537)
*Heir*	The dummy variable: this is ‘1’, if the landowner has an heir	0.4275 (0.4947)	0.2784 (0.4482)	0.1598 (0.3664)
*Main-Rice*	The dummy variable: this is ‘1’, if the landowner mainly cultivates rice	0.5645 (0.4958)	0.3830 (0.4861)	0.3051 (0.4605)
*Envfriend*	The dummy variable: this is ‘1’ if the landowner cultivates eco-friendly farming (decreasing chemical fertiliser)	0.2678 (0.4428)	0.2215 (0.4153)	0.09911 (0.2988)
*Machine*	The number of agricultural machines the landowner has	2.008 (1.386)	1.410 (1.512)	1.099 (1.548)
*Agr-Inc*	The income from agricultural products (JPY) (1 = ~15m, 2 = 15~30m, 3 = 30–60m, 4 = 60–90m, 5 = 90m~)	4.001 (2.594)	2.822 (2.925)	2.265 (2.903)
*Agr-CoInc*	The income from contract farming (JPY) (1 = ~15m, 2 = 15~30m, 3 = 30–60m, 4 = 60–90m, 5 = 90m~)	1.056 (0.6285)	0.7655 (0.7988)	0.6143 (0.8000)
*NoAgr-Inc*	The dummy variable: this is ‘1’ if non-agricultural income is higher than agricultural income	0.6259 (0.4839)	0.4083 (0.4915)	0.3028 (0.4595)
*Ship-Cons*	The dummy variable: this is ‘1’ if the landowner mainly ships products to consumers directly	0.05760 (0.2330)	0.06011 (0.2377)	0.04008 (0.1961)
*Fores-Prom*	The dummy variable: this is ‘1’ if the landowner lives in the forestry village area where public services were provided insufficient (i.e., Forestry Areas)	0.1543 (0.3612)	0.1644 (0.3707)	0.1625 (0.3689)
*Moun-Prom*	The dummy variable: this is ‘1’ if the landowner lives in the rural area which is mostly covered with steep area (i.e., Mountain Areas)	0.3082 (0.4617)	0.3264 (0.4689)	0.3264 (0.4689)
*Depo-Prom*	The dummy variable: this is ‘1’ if the landowner lives in the depopulated area (i.e., Depopulated Areas)	0.2378 (0.4257)	0.2749 (0.4465)	0.3258 (0.4687)
*Agri-Prom*	The dummy variable: this is ‘1’ if the landowner lives in Agriculture promotion area	0.9642 (0.1858)	0.9754 (0.1548)	0.9784 (0.1452)

### Ethics statement

Our dataset were provided by the MAFF; therefore, our dataset and study were not applicable to ethics approval.

### Econometric model

Based on a previous study that investigated the drivers affecting farmland abandonment (e.g., [27,42,54]), our study identifies the determinants of paddy land abandonment by focusing on socio-economic characteristics and geographical conditions. Paddy land owners decide how to use paddy land based on maximizing profits, including product prices and the condition of the paddy land [47,54]. Thus, higher paddy land productivity and higher agricultural income increase paddy land demand, which contributes to sustainable paddy land cultivation rather than abandonment. In addition, each rural community plays an important role in agriculture in Japan. For instance, irrigation systems and/or farm roads are managed by community members [55].

Since a substantial number of landowners exclusively cultivate paddy land, the dependent variables and areas of land abandonment are censored at zero as the minimum limit of the observable range [56]. If we apply the ordinary least squares method to censored data, the model predicts negative outcomes that may be inappropriate and inconsistent. In such situations, according to Wooldridge [57], the Tobit regression model is appropriate for censored data. Moreover, when a Tobit model is applied to panel data, it is better to use a random-effects model rather than a fixed-effects model (for details, see Cameron and Trivedi [58]). Hence, our data were applied to the random-effects Tobit model, which is described as:

$$y_{it}^{\prime }=x_{it}^{\prime }\;\beta +u_{it}\;u_{it}=\mu_{it}+\nu_{it}\;\nu_{\left\{it\right\}}∼iidN(0,\;\sigma_{v}^{2})$$

$$y_{it}=y_{it}^{\prime }\;if\;y_{it}^{\prime }>0$$

$$y_{it}=0\;if\;y_{it}^{*}\leq 0$$

where the area of paddy land in which individual *i* is abandoned in time period *t* is shown as *y*, and *y*′ and is an unobserved variable that satisfies the linear model assumptions; *x* is a vector of socio-economic characteristics, geographical conditions, and community relationships; *β* is a parameter vector to be estimated; *μ* is a time-invariant specific individual effect; and *v* is the remaining disturbance term.

If *μ* is independent of *x*, a random-effects model estimates the parameters. Assuming that *μ* follows $N(0,\;\sigma_{\mu }^{2})$, *v* also follows $N(0,\;\sigma_{v}^{2})$, and *μ* and *v* are independent, the log-likelihood function is:

$$\log\;L=\sum_{t=1}^{N}\log\;L_{i}$$

$$L_{i}=\int_{-\infty }^{\infty }\{\prod_{t=1}^{T_{i}}\left[\phi (\frac{-x_{it}^{\prime }\;\beta -\mu_{it}}{\sigma })][\frac{1}{\sigma_{v}}\phi (\frac{y-x_{it}^{\prime }\;\beta -\mu_{it}}{\sigma_{v}})\right]\}\phi (\frac{\mu_{i}}{\sigma_{\mu }})d\mu_{i}$$

Given that *μ* follows a normal distribution, the log-likelihood function is maximized through the integrals calculated using the Gauss-Hermite quadrature (for details, see Butler and Moffitt [59]). Parameters were estimated using ‘censReg packages’ in R [60,61]. In addition, we conducted a variance inflation factor (VIF) test for each model to avoid multicollinearity [62].

## Results

The results of the VIF test for each variable are less than 10 (see S1 Table). This indicates that none of the variables has potential multicollinearity problems [62]. Table 2 presents the estimated results are presented in Table 2; Model 1 is described in the left-hand columns of the table, whereas Model 2 is shown in the right-hand columns. The individual-specific effects are shown as the variable of log∑*μ*, and the variable of log∑*v* shows the remaining unexplained effects. The coefficients of these variables in both models were significant at the 0.1% level. Table 2 also shows that each coefficient had an effect on $y_{it}^{\prime }$, but not on *y*_*it*_. The log-likelihood value shows that Model 2 provides a better fit with the data than Model 1. We thus report the results of Model 2 and discuss the drivers in terms of the results of Model 2.

**Table 2:** Estimation results of the Tobit models.

Variables	Model 1		Model 2	
	Coefficient	S. E.	Coefficient	S. E.
*Own-Age*	-0.0760 ***	4.42 × 10^−3^	-0.0945 ***	4.55 × 10^−3^
*Own-Age* ^*2*^	0.351 × 10^−3^ ***	3.53 × 10^−5^	0.582 × 10^−3^ ***	3.62 × 10^−5^
*Own-Day*	-0.104 ***	3.48 × 10^−3^	-0.114 ***	3.60 × 10^−3^
*Workers*	-0.507 ***	5.24 × 10^−3^	-0.467 ***	5.47 × 10^−3^
*Pro-Fam*	0.543 ***	5.69 × 10^−3^	0.510 ***	5.89 × 10^−3^
*Ave-Age*	-0.405 ***	4.70 × 10^−3^	-0.375 ***	4.90 × 10^−3^
*Ave-Age* ^*2*^	3.66 × 10^−3^ ***	4.11 × 10^−5^	3.22 × 10^−3^ ***	4.26 × 10^−5^
*Yang*	-0.366 ***	7.24 × 10^−3^	-0.325 ***	7.41 × 10^−3^
*Woman*	-0.178 ***	0.0336	-0.0860 ***	0.0342
*Heir*	-1.11 ***	0.0139	-1.11 ***	0.0142
*Main-Rice*	-0.247 ***	0.0135	-0.233 ***	0.0138
*Envfriend*	-1.25 ***	0.0138	-1.31 ***	0.0141
*Machine*	0.0896 ***	4.58 × 10^−3^	0.138 ***	4.78 × 10^−3^
*Agr-Inc*	-0.946 ***	2.66 × 10^−3^	-0.812 ***	2.74 × 10^−3^
*Agr-CoInc*	-0.473 ***	4.22 × 10^−3^	-0.479 ***	4.31 × 10^−3^
*NoAgr-Inc*	0.812 ***	0.0155	0.748 ***	0.0161
*Ship-Cons*	0.826 ***	0.0264	0.812 ***	0.0268
*Fores-Prom*			−0.315 ***	0.0222
*Moun-Prom*			4.86 ***	0.0209
*Depo-Prom*			3.83 ***	0.0165
*Agri-Prom*			0.695 ***	0.0438
(Intercept)	1.71 ***	0.173	-2.45 ***	0.181
log∑*μ*	3.26 ***	0.195 × 10^−3^	3.24 ***	0.204 × 10^−3^
log∑*v*	2.42 ***	0.463 × 10^−5^	2.42 ***	0.481 × 10^−5^
Observations				
Total	4,770,108		4,592,658	
Zero-censored	3,867,164		3,716,762	
Log-likelihood	-7637895		-7377125	
Df	20		24	

***p < 0.001

**p < 0.01

*p < 0.05.

As shown in Table 2, seven variable coefficients are positive: the population of full-time cultivators in a household (*Pro-Fam*, coefficient in Model 1 and Model 2 is 0.544 and 0.510, respectively), the number of agricultural machines a landowner has (*Machine*, coefficient in Model 1 and Model 2 is 0.0902 and 0.138, respectively), non-agricultural income is higher than agricultural income (*NoAgr-Inc*, coefficient in Model 1 and Model 2 is 0.813 and 0.748, respectively), and the landowner directly ships products mainly to consumers (*Ship-Cons*, coefficient in Models 1 and 2 are 0.825 and 0.811, respectively). Paddy land location variables, except for Forest Areas, are also positive (S2 Table): the paddy land is located in the Mountain Areas (*Moun-Prom*, coefficient = 4.86), the paddy land is located in the Depopulated Areas (*Depo-Prom*, coefficient = 3.83), and the paddy land is located in an agricultural industry-promoting area (*Agri-Prom*, coefficient = 0.696). The variables of paddy landowners and household average age square are positive; however, the variables of paddy landowners and household average age are negative. Moreover, the coefficients of the other variables were significantly negative.

## Discussion and conclusion

Sustainable irrigated paddy land cultivation plays an essential role in biodiversity and ecosystem conservation in semi-natural Satoyama landscapes because the paddy land cultivation is a temporary artificial wetland [3]. However, farmland is being abandoned at an increasing rate worldwide, especially in developed countries [13,35], and biodiversity in and around paddy lands has also been decreasing [15]. This study identifies socio-economic drivers that affect individual landowners’ decisions to abandon irrigated paddy lands. These findings provide important insights into sustainable paddy land cultivation as a case study in the Asian monsoon climate region [13] and support effective policymaking to balance biodiversity and agroecosystem conservation in Satoyama landscapes.

Although irrigated paddy land is a unique cultivation system, most drivers affecting paddy land abandonment are similar to farmlands in other countries, such as northwestern Europe. Table 2 shows that paddy landowners with more successors and young people in their households tend not to abandon their paddy land. These results are also consistent with previous Japanese studies that have uncovered the determinants of farmland abandonment [42]. Moreover, Table 2 also shows that landowners who have more income from agricultural products tend not to abandon their paddy land. However, when non-agricultural income is higher than agricultural income, paddy landowners tend to abandon paddy land. This indicates that the lower the benefits from farming, the more paddy land is abandoned. These findings are similar to those of previous studies on farmland abandonment, particularly in Europe (e.g., [20,63,64]) and China (e.g., [25,26]). These results are also consistent with the theoretical framework of farmland abandonment developed based on the model of land sale and rental under the landowner’s profit maximization [47,54], and the findings concerning economic factors are also similar to the landowners’ motivation for biodiversity conservation on private land [65–67]. Therefore, the findings also support subsidy schemes such as direct payments to conserve paddy lands and the ecosystem services of paddy lands [68].

Our result also reveals that paddy land abandonment area does not likely increase monotonically with the age of paddy landowners and household average (Table 2), which supports previous findings in other countries [34] such as China [24,25] and Ghana [69]. Suppose that there is a threshold concerning landowners’ decision making on the paddy land abandonment depending on their age, it would be worth considering policy interventions targeting specific age groups. Up to the present, the effects of age on paddy land abandonment remain controversial in Japan. Some studies showed elderly paddy landowners tended to abandon their land because of part-time farmers (e.g., [27]), whereas other studies showed there was a possibility optimal age of paddy landowners to maintain paddy land cultivation (e.g., [47]). Effective policy design requires more detailed generation sensitivity analyses on landowner’s decision making on paddy land abandonment.

Our study contains interesting findings concerning the positive correlation between the population of full-time cultivators in a household and paddy land abandonment area, the results of which are different from previous Japanese studies [42]. It is difficult to explain this result, but it might be related to the dependent variable: the area of paddy land abandonment. Paddy landowners with many full-time cultivators can cultivate large paddy land areas before their retirement from cultivation. Thus, even though the ratio of abandoned paddy land area to cultivation area was small, a large paddy land area could be abandoned. These findings can provide new and useful insights for biodiversity conservation in semi-natural landscapes comprising paddy land.

Another interesting finding is that the proportion of women is an important driver of sustainable paddy land cultivation. Our analysis shows that the higher the ratio of women, the lower the area of abandoned land. This result is consistent with a previous study that uncovered a causal relationship between women’s participation in agricultural committees and farmland preservation in Japan [36]. This finding also supports the importance of women’s role not only in agriculture (e.g., [36–38]) but also in the conservation of agroecosystems and their services (e.g., [70–72]). Recently, gender equity has been recognized as fundamental to effective and sustainable efforts to stem biodiversity loss in the conservation field [70].

Table 2 shows that the amount of paddy land abandonment has a negative correlation with the variable of Forestry Areas despite it being a less-favored area. The farmland in the less-favored areas tends to be abandoned all over the world; however, our results implied that the area of paddy land abandonment in the Forestry Area can be lower than other areas, including the plain areas. Considering the positive relationship between the farmers in the Forestry Area and the receipt of direct payments [30], it seems possible that these results are due to the Japanese direct payment scheme that supports cultivation in the less-favored areas, despite the inclusion of non-target paddy land of the scheme in the area [32]. Thus, these findings partly support the previous studies, which reveal the positive impact of a direct payment scheme for maintaining farmland in the less-favored areas [30,43].

However, our analysis also confirms that paddy lands in less-favored areas tend to be abandoned except for the Forestry Areas. These findings are similar to those of previous studies, including Japanese studies [9,22,24,25,35,47,64]. Our results support Fukamachi, who pointed out the inadequacy of the Japanese scheme to enhance paddy land conservation [11]. In particular, the results indicate that it may be necessary to redesign the Japanese scheme to enhance paddy land conservation in less-favored areas apart from Forestry Areas (e.g., Mountain Areas and Depopulated areas). This is because the irrigated paddy land located in the cultivation of less-favored areas has multiple benefits in addition to the food supply, such as biodiversity conservation, beautiful landscape provisions, and the prevention of flood and soil erosion [3,10,11,18].

This study also had some limitations. Although the dataset derived from the Census of Agriculture and Forestry in Japan enables us to analyze substantial individual socio-economic panel data of irrigated paddy landowners, it did not include information about landowners’ attitudes and perceptions. Moreover, we were unable to access any qualitative data about paddy landowners. Given that considerable research on biodiversity conservation on private land indicates that landowners’ preferences are key factors (e.g., [73–75]), future research should address the impact of landowners’ preferences on paddy land maintenance and biodiversity conservation on irrigated paddy land. Another limitation is that we were not able to distinguish between biodiversity-rich paddy lands. Future research should investigate the relationship between paddy land cultivation and biodiversity-rich paddy lands to implement effective biodiversity conservation under budget limitations in Japan.

### Policy implications

Regarding the finding that plays a key role in preventing abandonment, it makes sense that recent Japanese agro-environmental policies are based on economic incentives for paddy land conservation [32]. In other words, a direct payment scheme can contribute to biodiversity conservation in semi-natural landscapes in Japan. This is because paddy land in the Forestry Area is also an important component of Satoyama landscapes [76]. Moreover, farmland cultivation enhances the management of forests, which are also a component of Satoyama landscapes [76,77]. Given that the variety of land use in Satoyama landscapes provides several types of habitats [77–80], it contributes to biodiversity conservation that Japanese agricultural measures to economically support cultivation in less-favored areas, such as a less-favored area direct payment scheme.

Paddy land tends to be abandoned, particularly in less-favored areas apart from the Forestry Areas. Thus, our findings also imply that economic incentives can still be insufficient to maintain irrigated paddy land cultivation in less-favored regions, although the prevention of paddy land abandonment is also affected by factors other than subsidies. In particular, there is still an increasing trend towards paddy land abandonment in Mountainous Areas. Considering the rich biodiversity and ecosystem services provided by features such as terraced paddy land, the government should enhance paddy land cultivation in these areas. Given our finding that higher income from agricultural products can prevent the abandonment of paddy lands, introducing a market-based approach can also enhance the conservation of multiple benefits from paddy lands. Previous studies have revealed that the market-based approach using wildlife labels is supported by consumers and generates higher revenue [40,41,81]. Moreover, environmentally-friendly farmers were negatively related to paddy land abandonment. This implies that the Japanese scheme concerning biodiversity-enhancing farming as a market-based approach makes sense in terms of biodiversity conservation and the prevention of land abandonment. A market-based approach can encourage biodiversity conservation through both production methods and paddy land cultivation. However, this only covers 0.07% of the cultivated rice in Japan [82]. Therefore, the government should regulate the market-based approach to prevent paddy land abandonment and promote biodiversity conservation on private land.

### Conclusion

Using an enormous set of individual panel data from the Japanese Census of Agriculture and Forestry, this study investigated the socio-economic determinants that influence irrigated paddy land cultivation sustainability in Japan, which is abandoned more than other types of farmlands such as drylands [42]. In this study, some determinants of paddy land abandonment are similar to those found in previous studies in other countries. For example, agricultural income correlates negatively with paddy land abandonment; however, there were several different effects on the drivers of abandonment. One is that paddy land in Forestry Areas tends not to be abandoned. It seems possible that these results are due to a Japanese direct payment scheme.

The findings of this research also provide several practical implications, such as the enhancement of a market-based approach to conserve the numerous benefits of paddy lands. This study also indicates the importance of socio-economic dimensions in developing biodiversity and agroecosystem conservation strategies. Considering that irrigated paddy land cultivation contributes to biodiversity [3–5,16,18], our findings on sustainable paddy land cultivation encourage effective policymaking to balance biodiversity conservation and the provision of agroecosystem services in semi-natural landscapes.

## Supporting information

S1 Table Results of the variance inflation factor tests for each variable. Note: Models 1 and 2 correspond to those in Table 2.
(DOCX)

S2 Table Estimation results in various favored area. (DOCX)
