# Supplementary material for: Socio-economic drivers of irrigated paddy land abandonment and agro-ecosystem degradation: Evidence from Japanese agricultural census data
Source: PLoS One. 2022 Apr 14;17(4):e0266997. doi: 10.1371/journal.pone.0266997 (PMC9009660; doi:10.1371/journal.pone.0266997)
Supplement: S1 Table — Note: Models 1 and 2 correspond to those in Table 2. (DOCX) [file pone.0266997.s001.docx]

**S1 Table. Results of the variance inflation factor tests for each variable**

| **Variables** | **Model 1** |  | | **Model 2** | |
| --- | --- | --- | --- | --- | --- |
| *Own-Age* | 1.428 | | 1.529 | |  |
| *Own-Day* | 1.944 | | 2.026 | |  |
| *Workers* | 2.220 | | 2.387 | |  |
| *Pro-Fam* | 1.316 | | 1.340 | |  |
| *Ave-Age* | 2.101 | | 2.104 | |  |
| *Yang* | 1.237 | | 1.251 | |  |
| *Woman* | 1.066 | | 1.084 | |  |
| *Heir* | 1.634 | | 1.657 | |  |
| *Main-Rice* | 1.381 | | 1.418 | |  |
| *Envfriend* | 1.257 | | 1.265 | |  |
| *Machine* | 1.847 | | 1.943 | |  |
| *Agr-Inc* | 1.817 | | 1.876 | |  |
| *Agr-CoInc* | 2.084 | | 2.201 | |  |
| *NoAgr-Inc* | 1.741 | | 1.805 | |  |
| *Ship-Cons* | 1.030 | | 1.032 | |  |
| *Fores-Prom* | - | | 2.729 | |  |
| *Moun-Prom* | - | | 2.704 | |  |
| *Depo-Prom* | - | | 1.851 | |  |
| *Agri-Prom* | - | | 1.034 | |  |

Note: Models 1 and 2 correspond to those in Table 2.
