# Supplementary material for: Socio-economic drivers of irrigated paddy land abandonment and agro-ecosystem degradation: Evidence from Japanese agricultural census data
Source: PLoS One. 2022 Apr 14;17(4):e0266997. doi: 10.1371/journal.pone.0266997 (PMC9009660; doi:10.1371/journal.pone.0266997)
Supplement: S2 Table — (DOCX) [file pone.0266997.s002.docx]

**S2 Table. Estimation results in various favored area**

| Variables | Forest Area | | |  | Mountain Area | | |  | Depopulated Area | |
| --- | --- | --- | --- | --- | --- | --- | --- | --- | --- | --- |
|  | Coefficient | | S. E. |  | Coefficient | | S. E. |  | Coefficient | S. E. |
| *Own-Age* | -0.0755 ^***^ | | 0.0159 | -0.0605 ^***^ | | 9.90 × 10^-3^ | | -0.0623 ^***^ | | 0.0120 |
| *Own-Age^2^* | 0.547 × 10^-3^ ^***^ | | 0.123 × 10^-3^ | 0.347 × 10^-3^ ^***^ | | 7.70 × 10^-5^ | | 0.306 × 10^-3^ ^***^ | | 9.30 × 10^-5^ |
| *Own-Day* | -0.341 ^***^ | | 0.0110 | -0.211 ^***^ | | 7.01 × 10^-3^ | | -0.135 ^***^ | | 8.71 × 10^-3^ |
| *Workers* | -0.756 ^***^ | | 0.0179 | -0.610 ^***^ | | 0.0106 | | -0.613^***^ | | 0.0146 |
| *Pro-Fam* | 0.879 ^***^ | | 0.0195 | 0.707 ^***^ | | 0.0124 | | 0.806 ^***^ | | 0.0151 |
| *Ave-Age* | -0.472 ^***^ | | 0.0141 | -0.441 ^***^ | | 9.35 × 10^-3^ | | -0.452 ^***^ | | 0.0112 |
| *Ave-Age^2^* | 3.90 × 10^-3^ ^***^ | | 0.119 × 10^-3^ | 3.74 × 10^-3^ ^***^ | | 7.95 × 10^-5^ | | 3.88 × 10^-3^ ^***^ | | 9.41 × 10^-5^ |
| *Yang* | -0.380 ^***^ | | 0.0244 | -0.373 ^***^ | | 0.0162 | | -0.291 ^***^ | | 0.0187 |
| *Woman* | 0.148 ^***^ | | 0.0905 | -0.315 ^***^ | | 0.0599 | | -0.442 ^***^ | | 0.0838 |
| *Heir* | -1.22 ^***^ | | 0.0450 | -1.19 ^***^ | | 0.0293 | | -1.23 ^***^ | | 0.0351 |
| *Main-Rice* | 0.541 ^***^ | | 0.0444 | 0.410 ^***^ | | 0.0288 | | -0.147 ^***^ | | 0.0342 |
| *Envfriend* | -1.83 ^***^ | | 0.0429 | -1.38 ^***^ | | 0.0273 | | -1.37 ^***^ | | 0.0319 |
| *Machine* | 0.0570 ^***^ | | 0.0167 | -0.0465 ^***^ | | 0.0103 | | 0.135 ^***^ | | 0.0126 |
| *Agr-Inc* | -0.971 ^***^ | | 8.89 × 10^-3^ | -0.842 ^***^ | | 5.60 × 10^-3^ | | -1.12 ^***^ | | 7.17 × 10^-3^ |
| *Agr-CoInc* | -0.0953 ^***^ | | 0.0315 | -0.151 ^***^ | | 0.0168 | | -0.228 ^***^ | | 0.0178 |
| *NoAgr-Inc* | 1.05 ^***^ | | 0.0536 | 1.01 ^***^ | | 0.0297 | | 1.09 ^***^ | | 0.0350 |
| *Ship-Cons* | -0.0809 ^***^ | | 0.0828 | -0.0679 ^***^ | | 0.0518 | | 0.338 ^***^ | | 0.0692 |
| (Intercept) | 5.58 ^***^ | | 0.559 | 4.95 ^***^ | | 0.360 | | 3.94 ^***^ | | 0.437 |
| $\log\sum\mu$ | 3.39 ^***^ | | 0.516 × 10^-3^ | 3.31 ^***^ | | 0.368 × 10^-3^ | | 3.41 ^***^ | | 0.380 × 10^-3^ |
| $\log\sum\nu$ | 2.59 ^***^ | | 1.51 × 10^-5^ | 2.51 ^***^ | | 0.932 × 10^-5^ | | 2.58 ^***^ | | 1.10 × 10^-5^ |
| Observations | |  | |  | | | |  | | |
| Total | | 722,102 | | 1,449,503 | | | | 1,235,684 | | |
| Zero-censored | | 545,381 | | 1,096,385 | | | | 938,597 | | |
| Log-likelihood | | -1500494 | | -2840935 | | | | -2391507 | | |
| Df | | 20 | | 20 | | | | 20 | | |
